# Postreplication Roles of the *Brucella* VirB Type IV Secretion System Uncovered via Conditional Expression of the VirB11 ATPase

**DOI:** 10.1128/mBio.01730-16

**Published:** 2016-11-29

**Authors:** Erin P. Smith, Cheryl N. Miller, Robert Child, Jennifer A. Cundiff, Jean Celli

**Affiliations:** a Paul G. Allen School for Global Animal Health, College of Veterinary Medicine, Washington State University, Pullman, Washington, USA; b Rocky Mountain Laboratories, National Institute for Allergy and Infectious Diseases, National Institutes of Health, Hamilton, Montana, USA

## Abstract

*Brucella abortus*, the bacterial agent of the worldwide zoonosis brucellosis, primarily infects host phagocytes, where it undergoes an intracellular cycle within a dedicated membrane-bound vacuole, the *Brucella*-containing vacuole (BCV). Initially of endosomal origin (eBCV), BCVs are remodeled into replication-permissive organelles (rBCV) derived from the host endoplasmic reticulum, a process that requires modulation of host secretory functions via delivery of effector proteins by the *Brucella* VirB type IV secretion system (T4SS). Following replication, rBCVs are converted into autophagic vacuoles (aBCVs) that facilitate bacterial egress and subsequent infections, arguing that the bacterium sequentially manipulates multiple cellular pathways to complete its cycle. The VirB T4SS is essential for rBCV biogenesis, as VirB-deficient mutants are stalled in eBCVs and cannot mediate rBCV biogenesis. This has precluded analysis of whether the VirB apparatus also drives subsequent stages of the *Brucella* intracellular cycle. To address this issue, we have generated a *B. abortus* strain in which VirB T4SS function is conditionally controlled via anhydrotetracycline (ATc)-dependent complementation of a deletion of the *virB11* gene encoding the VirB11 ATPase. We show in murine bone marrow-derived macrophages (BMMs) that early VirB production is essential for optimal rBCV biogenesis and bacterial replication. Transient expression of *virB11* prior to infection was sufficient to mediate normal rBCV biogenesis and bacterial replication but led to T4SS inactivation and decreased aBCV formation and bacterial release, indicating that these postreplication stages are also T4SS dependent. Hence, our findings support the hypothesis of additional, postreplication roles of type IV secretion in the *Brucella* intracellular cycle.

## IMPORTANCE

Many intracellular bacterial pathogens encode specialized secretion systems that deliver effector proteins into host cells to mediate the multiple stages of their intracellular cycles. Because these intracellular events occur sequentially, classical genetic approaches cannot address the late roles that these apparatuses play, as secretion-deficient mutants cannot proceed past their initial defect. Here we have designed a functionally controllable VirB type IV secretion system (T4SS) in the bacterial pathogen *Brucella abortus* to decipher its temporal requirements during the bacterium’s intracellular cycle in macrophages. By controlling production of the VirB11 ATPase, which energizes the T4SS, we show not only that this apparatus is required early to generate the *Brucella* replicative organelle but also that it contributes to completion of the bacterium’s cycle and bacterial egress. Our findings expand upon the pathogenic functions of the *Brucella* VirB T4SS and illustrate targeting of secretion ATPases as a useful strategy to manipulate the activity of bacterial secretion systems.

## INTRODUCTION

Intracellular Gram-negative bacterial pathogens have the capacity to subvert host cell functions and generate or reach compartmentalized niches that provide them with survival, persistence, and proliferation abilities essential to their virulence. They achieve these pathogenic feats via delivery of effector proteins through dedicated secretion systems that are key to their virulence (1). By delivering an array of effectors involved in modulating multiple host functions, these secretion systems often contribute to distinct, sequential stages of bacterial intracellular cycles. Classical genetic, mutant-based approaches designed to determine their roles can, however, reveal their functions only in the earliest stage that they control, as secretion-deficient mutants cannot proceed past their initial defect (2–4). These limitations have generally restricted our understanding of the role secretion systems play in late stages of the pathogens’ intracellular life cycles (5, 6).

Bacteria of the genus *Brucella* are the causative agents of brucellosis, a zoonosis of global importance that causes abortion and sterility in their primary animal hosts and a febrile recurrent chronic illness in humans following accidental exposure and infection through mucosal surfaces (7). The ability of *Brucella* spp. to cause disease depends on their intracellular cycle within host phagocytes, such as macrophages or dendritic cells (8, 9), in which the bacterium resides in a membrane-bound compartment called the *Brucella*-containing vacuole (BCV) (10). Upon their initial formation following phagocytic uptake, BCVs undergo maturation events along the endocytic pathway to become an endosomal BCV (eBCV) (10–12). eBCVs feature an acidic pH and hydrolytic activity and accumulate late endocytic/lysosomal markers on their vacuolar membrane, such as lysosome-associated membrane protein 1 (LAMP1), indicative of a lysosomal nature (13, 14). eBCVs then interact with endoplasmic reticulum (ER) exit sites (ERES) along the host early secretory pathway and are progressively remodeled into replication-permissive vacuoles (rBCVs) derived from, and with functional properties of, the ER (10, 11, 15), where *Brucella* undergoes replication. Subsequent to bacterial proliferation, rBCVs are engulfed into autophagosome-like structures to become autophagic BCVs (aBCVs), a final stage that facilitates completion of the bacterium’s intracellular cycle by promoting bacterial egress (12). *Brucella* therefore displays a multistage intracellular cycle, which illustrates the complexity of its interactions with host cellular pathways and suggests that the bacterium actively controls these sequential intracellular events.

Remodeling of eBCV to rBCV requires functions of the *Brucella* VirB type IV secretion system (T4SS), a protein translocation apparatus essential for intracellular replication, modulation of host immune functions, and establishment of chronic brucellosis (7, 16), as mutants of various *virB* genes encoding T4SS components are defective in rBCV biogenesis and replication (2, 10, 17), stalling in eBCVs, where they are eventually killed (10). While the dependence of rBCV biogenesis on VirB argues that this stage of the *Brucella* intracellular cycle is mediated by the action of bacterially delivered effectors, it is unknown whether replication in rBCV and postreplication stages, such as aBCV formation and bacterial egress, are VirB dependent. Since VirB-deficient strains of *Brucella* do not reach the replicative rBCV stage, genetic inactivation of the VirB apparatus cannot be used to examine whether any stage post-rBCV biogenesis is a VirB T4SS-dependent process. We reasoned that temporal control of VirB functionality during the bacterium’s intracellular cycle may address these issues. Here we have designed and generated strains of *Brucella abortus* in which expression of an essential *virB* gene depends upon the presence of the inducer anhydrotetracycline (ATc) and can be induced or repressed in a temporal manner to control functionality of the VirB T4SS. Applying this approach to the VirB11 ATPase, we show that VirB T4SS expression within the eBCV is required early for optimal rBCV biogenesis and is also necessary for aBCV formation and bacterial egress, therefore uncovering novel roles of this secretion machinery in postreplication stages of the *Brucella* intracellular cycle.

## RESULTS

### Inactivation and conditional complementation of the *B. abortus* VirB11 ATPase.

**FIG 1:**
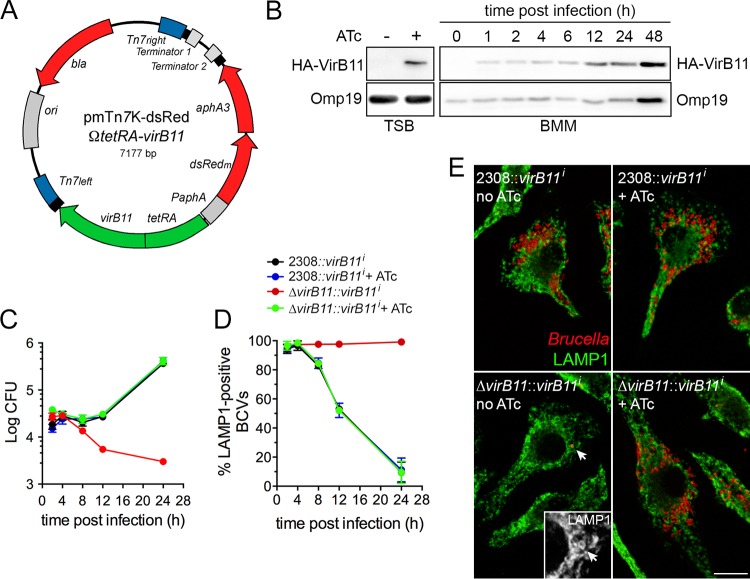
Conditional complementation of a *B. abortus* Δ*virB11* mutant during intracellular trafficking and growth in BMMs. (A) Map of the mini-Tn*7*K-dsRedΩ*tetRA-virB11* transposon delivery plasmid for ATc-dependent complementation of the *virB11* deletion. The *P_aph_-dsRed_m_-aphA3* (red) and *tetRA-virB11* (green) regions were cloned in divergent orientations to avoid transcriptional readthrough of *virB11*. Tn*7* ends (blue) and transcriptional terminators are indicated. (B) Representative induction of HA-VirB11 production upon ATc treatment in either tryptic soy broth (TSB) or macrophages (BMM). Western blots were probed with either anti-HA for detection of VirB11 or anti-*Brucella* Omp19 antibodies as a loading control. (C) Intracellular replication in BMMs of either *B. abortus* 2308::*virB11i* or 2308Δ*virB11*::*virB11i* strains in the presence or absence of ATc induction. Data are means ± SD of results of 3 independent experiments. (D) Intracellular trafficking of BCVs containing either *B. abortus* 2308::*virB11i* or 2308Δ*virB11*::*virB11i* strains in the presence or absence of ATc induction, measured via acquisition (eBCV) and then exclusion (rBCV) of the late endosomal/lysosomal marker LAMP1. Data are means ± SD of results of 3 independent experiments. (E) Representative confocal micrographs of BMMs infected for 24 h with either *B. abortus* 2308::*virB11i* or 2308Δ*virB11*::*virB11i* strains in the presence or absence of ATc induction. Bacteria (red) replicate within LAMP1-negative compartments, except for the conditional mutant 2308Δ*virB11*::*virB11i* in the absence of ATc. Scale bar, 10 µm.

To assess the role of the VirB T4SS in postreplication stages of the *Brucella* intracellular cycle, we reasoned that enabling VirB T4SS activity to allow rBCV biogenesis and then disabling its function to test its role in bacterial replication within rBCVs, aBCV formation, and bacterial egress would circumvent the early trafficking blockage that characterizes constitutively defective *virB* mutant strains in which single or multiple *virB* genes are inactivated (2, 10, 15, 18). We therefore sought to generate a *B. abortus* strain in which VirB T4SS activity is conditional by constructing an in-frame deletion mutant in an essential *virB* gene and complementing it with a conditionally expressed, single chromosomal copy inserted at a secondary locus. The *Brucella* VirB T4SS is composed of 11 VirB subunits (VirB1 to VirB11). On the basis of their homology with other closely related P-type VirB T4SSs, such as those of *Agrobacterium tumefaciens* or the IncW conjugative plasmid R388, VirB2, VirB3, VirB5, VirB6, VirB7, VirB8, VirB9, and VirB10 subunits likely play structural roles (19), while VirB4 and VirB11 are secretion ATPases that energize the system for T4SS assembly and effector translocation (19). We chose to target the VirB11 ATPase, based on (i) the location of *virB11* as the last gene of the *virB* operon, to avoid polar effects of its deletion; (ii) its demonstrated role in VirB T4SS-dependent functions (2); and (iii) its turnover being potentially faster than that of VirB structural components, as an inner membrane-associated ATPase (19). We first generated an in-frame deletion of codons 6 to 356 of *virB11* via SacB-assisted allelic replacement (15) to generate *B. abortus* 2308Δ*virB11*. We then constructed a derivative of a *Brucella*-adapted mini Tn*7* transposon (20) carrying two different cistrons (Fig. 1A): the hemagglutinin (HA)-tagged *virB11* gene under the control of the *tetR-P*_*tetA*_ region of Tn*10* to synthesize HA-VirB11 in an anhydrotetracycline (ATc)-dependent manner and an artificial *dsRed_m_-aphA3* bicistronic operon under the control of the constitutive *P*_*aphA3*_ promoter for kanamycin selection and fluorescence microscopy purposes. The resulting transposon, mTn*7*K-*tetRA-virB11* (Fig. 1A), was inserted at the single *att* Tn*7* locus downstream of *glmS* in *B. abortus* 2308Δ*virB11* to generate the VirB conditional strain *2308*Δ*virB11*::*virB11i*. The same transposon was also inserted into the wild-type strain to generate *B. abortus 2308*::*virB11i* and to test for any deleterious effect of a second chromosomal copy of *virB11*. ATc control of VirB11 production in strain *2308*Δ*virB11*::*virB11i* was first tested in tryptic soy broth (TSB), and the results showed no detectable production of VirB11 in the absence of ATc and clear induction after 4 h of treatment with ATc (100 ng/ml) (Fig. 1B, left panel), indicating that VirB11 production is controllable by ATc. We then tested the intracellular behavior of both strain *2308*::*virB11i* and strain *2308*Δ*virB11*::*virB11i* in murine bone marrow-derived macrophages (BMMs) in the presence and absence of ATc. Intracellular growth of strain *2308*::*virB11i* showed a typical intracellular growth pattern regardless of the presence or absence of ATc, with an increase in recoverable bacterial numbers after 12 h pi (Fig. 1C), indicating that the presence of the inducible chromosomal copy of *virB11* does not affect *Brucella* intracellular growth. In the absence of ATc, the *2308*Δ*virB11*::*virB11i* strain showed the defective intracellular growth typical of a VirB-deficient mutant (2, 10, 15), indicating tight repression of VirB11 production by the *tetR-P*_*tetA*_ region. In contrast, addition of ATc at the time of infection restored intracellular growth of the VirB conditional strain to wild-type levels, demonstrating full complementation of the *virB11* deletion by the ATc-inducible copy of *virB11*. Under these experimental conditions, VirB11 was detected by Western blotting as early as 1 h postinfection (pi) and throughout a 48 h time course during which ATc treatment was maintained (Fig. 1B, right panels), increasing with bacterial replication past 12 h pi. This demonstrated that VirB11 synthesis in strain *2308*Δ*virB11*::*virB11i* is maintained by prolonged ATc induction. Consistent with intracellular growth, monitoring BCV intracellular trafficking during the first 24 h pi through acquisition (eBCV stage) and then exclusion (rBCV stage) of lysosomal membrane marker LAMP1 on BCV membranes (13) showed a drastic defect in rBCV biogenesis in the *2308*Δ*virB11*::*virB11i* strain in the absence of ATc, in agreement with a VirB functional defect, which was fully complemented upon ATc addition (Fig. 1D). Confocal microscopy analysis of infected BMMs at 24 h pi confirmed that ATc-treated BMMs infected with strain *2308*Δ*virB11*::*virB11i* harbored replicating intracellular bacteria within LAMP1-negative rBCVs at levels similar to those seen with the ATc-treated or untreated *2308*::*virB11i* control strain, while strain *2308*Δ*virB11*::*virB11i* did not show any replication pattern and was restricted to LAMP1-positive eBCVs in the absence of ATc (Fig. 1E), as expected of VirB-deficient bacteria. Taken together, these results establish that we have generated a *B. abortus* strain in which VirB functionality can be tightly controlled intracellularly using ATc, allowing us to switch from VirB-deficient to VirB-competent bacteria.

### VirB T4SS function is required early during infection for optimal intracellular replication.

**FIG 2:**
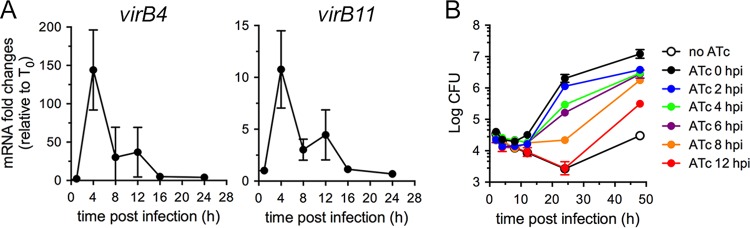
VirB T4SS function is required early during infection for optimal intracellular replication. (A) Representative profiles of *virB4* and *virB11* expression measured by quantitative PCR of their mRNAs during infection of BMMs with wild-type *B. abortus* strain 2308. Data are means ± SD of results of 3 independent experiments performed in triplicate and are expressed as fold changes compared to samples collected at the time of infection (T0). (B) Intracellular growth of *B. abortus* strain 2308Δ*virB11*::*virB11i* in BMMs upon addition of ATc (100 ng/ml) at various times pi, measured via enumeration of CFUs over 48 h. Data are means ± SD of results of 3 independent experiments performed in triplicate.

On the basis of the characterization presented above, we first sought to determine the temporal requirements of VirB functions during the *Brucella* intracellular cycle. Early defects of VirB-deficient mutants, which are restricted to eBCVs (2, 10), and previous analysis of intracellular *virB* gene expression argue for a rapid induction of the VirB T4SS (13, 21, 22), suggesting that its activity is required early during the bacterium’s intracellular cycle. Yet this assumption has not been formally demonstrated. We first examined intracellular expression of the *virB* operon under our experimental conditions in wild-type *B. abortus* 2308 via quantitative PCR (qPCR) using either *virB4* or *virB11* mRNAs and confirmed very strong induction upon bacterial uptake by BMMs which peaked at 4 h pi (~150-fold for *virB4*; ~12-fold for *virB11*) and decreased thereafter (Fig. 2A), consistent with previous reports (13, 21, 22). To determine VirB T4SS functional requirements at these time points, we infected BMMs with strain *2308*Δ*virB11*::*virB11i* and induced VirB activity with sequential delays postinfection to monitor bacterial growth over time. Compared to ATc induction at the time of infection (0 h pi), induction at 2 h pi did not significantly affect bacterial replication (Fig. 2B). In contrast, ATc addition at either 4 or 6 h pi decreased bacterial replication at 24 h pi by nearly 1 order of magnitude (Fig. 2B). Moreover, ATc addition at 8 h pi decreased replication by 2 orders of magnitude, yielding no net increase in bacterial numbers by 24 h pi (Fig. 2B). ATc addition at 12 h pi failed to mediate any growth of strain *2308*Δ*virB11*::*virB11i*, which behaved similarly to the non-ATc-induced, VirB-deficient bacteria at 24 h pi (no ATc; Fig. 2B). While bacteria treated with ATc from 0 to 6 h pi sustained similar growth after 24 h pi, those treated at either 8 or 12 h pi underwent substantial replication between 24 and 48 h pi (Fig. 2B) and yet did not reach control levels (ATc; 0 h pi), indicating that the growth defects seen at 24 h pi upon delayed VirB induction were recoverable. Of note, minor bacterial growth was observed between 24 and 48 h pi in the absence of ATc induction (no ATc), which reflected limited replication in a few BMMs (data not shown), possibly due to leaky *virB11* expression in these bacteria. Altogether, this demonstrates that the *Brucella* VirB T4SS is functionally required early during the bacterium’s intracellular cycle, consistent with its intracellular expression pattern, and that its function needs temporal coordination with the stage of conversion of eBCV to rBCV to mediate optimal initiation of intracellular growth.

### Minimal induction of VirB11 required for rBCV biogenesis and replication.

**FIG 3:**
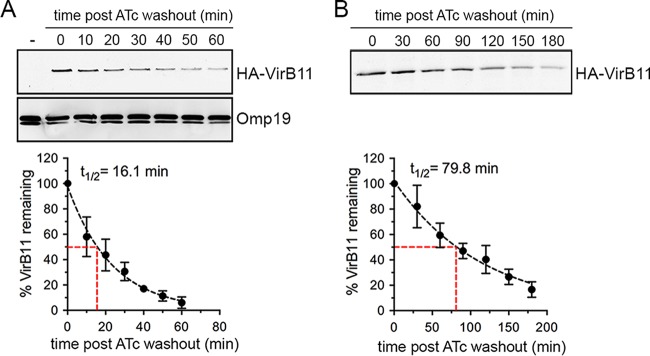
The VirB11 ATPase displays differential stability during culture and infection. (A) Representative results of Western blot analysis of HA-VirB11 produced by *B. abortus* strain 2308Δ*virB11*::*virB11i* following 4 h of ATc induction in TSB, at various time points post-ATc washout and chloramphenicol addition, and calculation of its half-life (t_1/2_) based on results of 3 independent experiments. Omp19 detection was used as a loading control. (B) Representative Western blot analysis of HA-VirB11 produced by *B. abortus* strain 2308Δ*virB11*::*virB11i* following a 6 h ATc induction in BMMs, at various time points post-ATc washout and chloramphenicol addition, and calculation of its half-life based on results of 3 independent experiments. Samples were loaded according to equivalent CFU counts.

**FIG 4:**
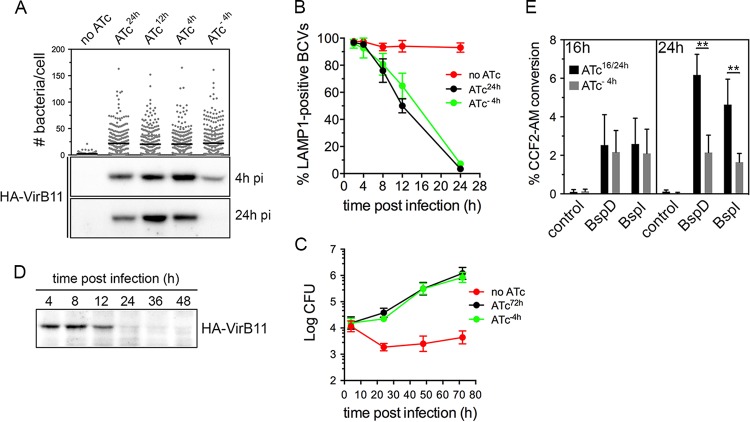
Minimal induction of VirB11 required for rBCV biogenesis and replication. (A) Intracellular replication of, and HA-VirB11 production at either 4 or 24 h pi in, *B. abortus* strain 2308Δ*virB11*::*virB11i* following ATc treatments for 24 h (ATc^24h^), 12 h (ATc^12h^), or 4 h postinfection (ATc^4h^) or following 4 h of ATc preinduction (ATc^−4h^). Intracellular replication was measured as numbers of bacteria/BMM at 24 h pi. Data are means ± SD of results from 3 independent experiments. (B) Intracellular trafficking of BCVs containing *B. abortus* 2308Δ*virB11*::*virB11i* in the absence of ATc treatment (no ATc) or following sustained ATc treatment (ATc^24h^) or following 4 h of ATc preinduction (ATc^−4h^). Data are means ± SD of results of 3 independent experiments. (C) Intracellular replication in BMMs of *B. abortus* 2308Δ*virB11*::*virB11i* strains in the absence of ATc treatment (no ATc) or following sustained ATc treatment (ATc^24h^) or following 4 h of ATc preinduction (ATc^−4h^). Data are means ± SD of results of 3 independent experiments performed in triplicate. (D) Representative Western blot analysis of HA-VirB11 decay during BMM infection with *B. abortus* strain 2308Δ*virB11*::*virB11i* following 4 h of ATc preinduction (ATc^−4h^). Samples were loaded based on equivalent CFU counts. (E) TEM1 β-lactamase translocation reporter assay of VirB T4SS activity in *B. abortus* strain 2308Δ*virB11*::*virB11i* either upon sustained ATc treatment (ATc^16/24h^) or following 4 h of ATc preinduction (ATc^−4h^). Translocation of C-terminal TEM1 fusions to effector BspD or BspI or to negative-control *B. abortus* protein BAB2_0654 was measured via fluorescence microscopy analysis of CCF2-AM cleavage at either 16 or 24 h pi in J774.A1 cells. Data are means ± SD of results of 3 independent experiments performed in triplicate. **, statistically significant difference (two-way ANOVA followed by Bonferroni’s multiple-comparison test; *P* < 0.01).

On the basis of the effective control of VirB T4SS activity through conditional production of VirB11, in order to examine potential roles of the VirB T4SS in postreplication stages of the *Brucella* intracellular cycle, we next sought to determine whether this system is amenable to inactivation once the VirB T4SS has been induced. We reasoned that inactivation of the VirB T4SS requires turnover of HA-VirB11 sufficient to render the T4SS inactive and therefore first examined HA-VirB11 stability upon ATc removal. Following a 4-h induction with ATc in TSB, ATc washout, and addition of chloramphenicol (Cm; 10 µg/ml) to inhibit *de novo* protein synthesis, HA-VirB11 displayed a progressive decrease within a 60-min time course analysis, with a calculated half-life (*t*_1/2_) of 16.1 min (Fig. 3A), indicating a significant instability of the protein. Upon infection of BMMs with strain *2308*Δ*virB11*::*virB11i* and ATc induction for 6 h, however, HA-VirB11 displayed a longer half life (*t*_1/2_ = 79.8 min; Fig. 3B), reflecting increased stability in intracellular bacteria. Yet these results suggested that the turnover of HA-VirB11 that occurred was sufficient to inactivate VirB T4SS-dependent functions past 24 h pi. On the basis of these results, we aimed to define the minimal induction conditions that would mediate rBCV biogenesis and *Brucella* replication by 24 h pi and yet lead rapidly thereafter to VirB T4SS inactivation via HA-VirB11 turnover. We therefore compared the levels of bacterial replication of strain *2308*Δ*virB11*::*virB11i* in BMMs that occurred upon ATc treatment at 24 h (ATc^24h^), 12 h (ATc^12h^), and 4 h (ATc^4h^) pi and 4 h prior to infection (ATc^−4h^). Surprisingly, all treatments supported comparable growth levels by 24 h pi (Fig. 4A), indicating that a short (4 h) period of induction in bacterial culture prior to infection (ATc preinduction) was sufficient to provide strain *2308*Δ*virB11*::*virB11i* with the VirB-dependent functions necessary to generate rBCVs and initiate replication. To confirm these results, we compared levels of rBCV biogenesis and bacterial growth of strain *2308*Δ*virB11*::*virB11i* under conditions of either sustained ATc treatment (ATc^24h^) or 4 h treatment prior to infection (ATc^−4h^). Compared to the results seen in the absence of ATc induction, ATc preinduction was sufficient to restore rBCV biogenesis and bacterial replication to the levels seen with the sustained induction at up to 72 h pi (Fig. 4B and C). Importantly, HA-VirB11 was detectable by Western blotting at 4 h pi under all conditions but not by 24 h pi upon ATc preinduction (Fig. 4A). This indicates that ATc preinduction led to significant HA-VirB11 turnover by the time bacteria replicated in rBCVs, suggesting VirB T4SS inactivation by this stage. Further examination of intracellular HA-VirB11 turnover in BMMs upon 4 h of ATc preinduction showed it was no longer detectable after 12 h pi (Fig. 4D), further suggesting that these experimental conditions are amenable to the analysis of the role of the VirB T4SS in postreplication stages. To confirm this, we evaluated VirB T4SS functionality by measuring accumulated delivery of known *Brucella* effectors, BspD and BspI (20, 23), into J774A.1 macrophages via a TEM1 β-lactamase translocation assay. Compared to TEM1 fusion to a *B. abortus* protein (encoded by the BAB2_0654 locus) that is not translocated upon infection (20), at 16 h pi, BspD and BspI showed significant and comparable levels of translocation from strain *2308*Δ*virB11*::*virB11i* bacteria treated with ATc either for the whole infection time (ATc^16h^) or for a 4 h preinduction (ATc^−4h^) (Fig. 4E). This argues for the occurrence of similar levels of VirB T4SS activity under the two sets of ATc conditions at this time point. While accumulation of translocated BspD and BspI in J774A.1 cells between 16 and 24 h pi upon sustained ATc treatment (ATc^16/24h^) was detectable via an increase in the percentage of cells where CCF2-AM conversion occurred, their delivery by *2308*Δ*virB11*::*virB11i* bacteria subjected to the 4 h preinduction (ATc^−4h^) was stalled between these time points (Fig. 4E), indicating a loss of VirB T4SS activity between 16 and 24 h pi that is consistent with the decay of HA-VirB11 observed under the same experimental conditions (Fig. 4D). These differences in T4SS effector delivery did not result from differences in viable intracellular bacteria, as the two ATc treatments yielded comparable intracellular CFU levels during the time frame of analysis (Fig. 4C). This demonstrates a strongly reduced function of the VirB T4SS during the bacterial replication phase in rBCVs under conditions of minimal induction of HA-VirB11 prior to infection. Taken together, these results established experimental conditions that allow (i) VirB-dependent early stages of the *Brucella* intracellular cycle to proceed and (ii) subsequent reduction of VirB T4SS activity, thus permitting analysis of its role in *Brucella* postreplication stages.

### Inactivation of the VirB T4SS postreplication impairs aBCV formation and bacterial release.

**FIG 5:**
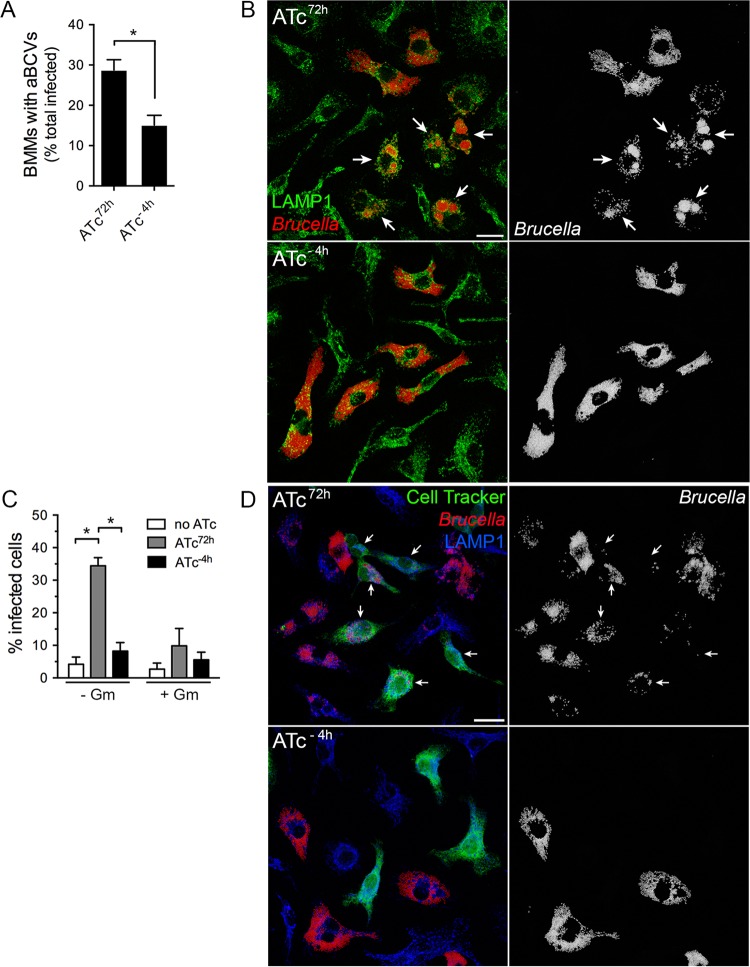
Inactivation of the VirB T4SS postreplication impairs aBCV formation and bacterial release. (A) Quantification of aBCV formation in BMMs infected with *B. abortus* 2308Δ*virB11*::*virB11i* either upon sustained ATc treatment (ATc^72h^) or following 4 h of ATc preinduction (ATc^−4h^). Data represent the percentages of infected BMMs containing aBCVs at 72 h pi and are means ± SD of results of 3 independent experiments. (B) Representative confocal micrographs of aBCV formation in BMMs infected with *B. abortus* 2308Δ*virB11*::*virB11i* either upon sustained ATc treatment (ATc^72h^) or following 4 h of ATc preinduction (ATc^−4h^). Arrows in both the composite (LAMP1 in green; *Brucella* in red) and single-channel (*Brucella*) images show aBCV-containing BMMs. Scale bar, 20 µm. (C) Quantification of reinfection events in BMMs infected with *B. abortus* 2308Δ*virB11*::*virB11i* either upon sustained ATc treatment (ATc^72h^) or following 4 h of ATc preinduction (ATc^−4h^). Unlabeled BMMs were infected, and a secondary cell Tracker Green-labeled BMM population was added at 44 h pi and monitored for infection between 48 and 72 h pi in the presence or absence of gentamicin (Gm; 20 µg/ml). Data represent the percentages of Cell Tracker Green-labeled BMMs that contained bacteria at 72 h pi and are means ± SD of results of 3 independent experiments. *, statistically significant difference (two-way ANOVA followed by Sidak’s multiple-comparison test; *P* < 0.05). (D) Representative confocal micrographs of reinfection events in BMMs infected with *B. abortus* 2308Δ*virB11*::*virB11i* either upon sustained ATc treatment (ATc^72h^) or following 4 h of ATc preinduction (ATc^−4h^). Arrows in both the composite and single-channel (*Brucella*) images indicate Cell Tracker Green-labeled BMMs containing bacteria. Scale bar, 20 µm.

Subsequent to VirB T4SS-dependent rBCV biogenesis, *Brucella* undergoes replication that is followed by rBCV remodeling into aBCVs (24 to 72 h pi), a process that facilitates bacterial egress (12). Whether replication, aBCV formation, and bacterial egress are VirB-dependent processes is unknown. On the basis of the detection of reduced VirB activity by 24 h pi following the 4 h ATc preinduction treatment (Fig. 4), we first sought to test whether *Brucella* intracellular growth was affected between 24 and 72 h pi in BMMs. Compared to sustained ATc treatment, early transient activation of the VirB T4SS did not affect *Brucella* replication (Fig. 4C), indicating either that a functional T4SS is dispensable for growth within rBCVs or that its functional requirements are established prior to 24 h pi and sustained over time. In contrast, formation of aBCVs was significantly reduced (28.8% ± 2.5% of infected BMMs under conditions of sustained activation compared to 15.1% ± 2.4% under conditions of transient activation, *P* = 0.0024 [*t* test]; Fig. 5A and B). Bacterial egress was then assessed by adding a secondary population of Cell Tracker Green-labeled BMMs to the originally infected, unlabeled BMMs and by measuring their infection between 48 and 72 h pi upon removal of gentamicin. The percentage of newly infected, Cell Tracker Green-positive BMMs was dramatically decreased upon inactivation of the VirB T4SS (34.5% under conditions of sustained activation compared to 5.5% under conditions of transient activation; Fig. 5C and D) to the level seen with sustained treatment with gentamicin (Fig. 5C; see also Fig. S1 in the supplemental material), which blocked reinfection events (12). Considering that aBCVs facilitate *Brucella* release from macrophages (12), these findings clearly demonstrate that aBCV formation and subsequent bacterial egress are VirB T4SS-dependent processes.

## DISCUSSION

Secretion systems encoded by intracellular bacteria deliver arrays of effectors that modulate host cell processes involved in multiple stages of their intracellular cycles, including invasion, modulation of phagosome maturation, inhibition of innate immune responses, biogenesis of replicative organelles, and bacterial egress. Because these intracellular events occur sequentially, classical genetic, mutant-based approaches have failed to address the roles that secretion systems play in later stages, as secretion-deficient mutants cannot proceed past their initial defect. *Brucella* spp. undergo a complex intracellular cycle in phagocytes which invokes the sequential modulation of host endocytic, secretory, and autophagic machineries (16). While the role of the VirB T4SS in rBCV biogenesis has been clearly established via the use of VirB-defective mutants (2, 10, 15), its temporal requirements and roles in postreplication stages eluded examination until the functionality of the VirB T4SS apparatus could be temporally controlled.

A few studies have previously used postentry inactivation approaches with other pathogens. Arabinose-inducible control of the *Shigella flexnerii* Mxi-Spa type III secretion system gene *icsA* has revealed a postinvasion role of the *S. flexnerii* Mxi-Spa type III secretion system in the bacterium’s intercellular spread (5). Inducible control of Cre recombinase-mediated deletion of *Legionella pneumophila* Dot/Icm T4SS gene *icmQ*, and of T4SS effector *sdhA*, has highlighted their roles in intracellular bacterial replication (6). Previous evidence has, however, suggested that T4SSs may remain stable once assembled and active even after downregulation of their expression (6, 24), questioning the possibility of controllable inactivation of a T4SS. In our attempt to modulate the activity of the VirB T4SS by controlling production of an essential VirB component, we chose to target the VirB11 ATPase, given its predicted peripheral association with the apparatus at the inner membrane (19) and its role in providing energy for T4SS effector translocation. As such, we reasoned that depletion of VirB11 would inactivate the VirB T4SS, even if structural VirB components are stably assembled, a scenario supported by the previous demonstration that loss of VirB11 production via deletion of the *virB11* gene does not affect production or stability of other VirB components (17). Complementation of a *virB11* in-frame deletion with an ATc-controllable chromosomal copy of *virB11* showed tight control of VirB11 synthesis in the absence of induction and full conditional complementation of the mutant. Such tight transcriptional control was important to clearly demonstrate the temporal requirements of VirB T4SS activity during conversion of eBCV to rBCV, without which bacteria displayed protracted growth, even after ATc-mediated induction of VirB11. This temporal coordination for VirB T4SS requirements is consistent with the natural expression pattern of the *virB* operon (13, 21), whose expression is rapidly induced in eBCVs, peaks during rBCV biogenesis, and progressively decreases at the onset of bacterial replication. While such an expression pattern may indicate a requirement of the T4SS only at early stages, it may also reflect rapid production as well as assembly that is prolonged over time to fulfill additional functions at later stages of the bacterium’s cycle.

Assessing late roles of the VirB T4SS requires early activation of the VirB T4SS to allow rBCV biogenesis and the onset of bacterial replication, followed by a rapid turnover of VirB11 upon repression of its production. While HA-tagged VirB11 showed rapid turnover in broth culture, the rate was considerably lower in intracellular bacteria. The cause of the observed differences in the levels of VirB11 stability under broth and intracellular conditions is unclear, but the differences could either result from the experimental conditions or be biologically relevant: The efficiency of intracellular TetR-mediated repression of *virB11* expression may not have been as high as that seen in broth, due to a longer persistence of ATc in intracellular bacteria after washout that would have prolonged VirB11 synthesis. Yet this should not have affected the outcome of chloramphenicol chase experiments, where *de novo* protein synthesis is blocked. Hence, we favor a biological explanation, where the VirB11 ATPase is stabilized when engaged in an actively translocating T4SS in intracellular bacteria compared to broth culture conditions where VirB components are produced but may not be assembled or engaged in effector translocation. Future studies are needed to address this issue. Regardless of the increased stability of VirB11 in mammalian cells, its intracellular turnover was sufficient to abolish its detectable expression between 12 and 24 h pi upon a short period of ATc induction prior to infection, consistent with its dilution during the bacterial division initiated between these time points. Loss of VirB11 production in intracellular bacteria was corroborated by a block in accumulation of effector translocation between 16 and 24 h pi, arguing for VirB T4SS inactivation during the early stages of bacterial replication. On the basis of these results, we did not see any defect in further bacterial replication between 24 and 72 h pi, suggesting that the VirB T4SS is not required for bacterial growth once bacteria have established their rBCV. However, we cannot exclude the possibility that some VirB effectors are required for bacterial growth within rBCVs but were delivered prior to VirB inactivation under our experimental conditions, i.e., before 24 h pi, and remained active afterward. Hence, future studies will aim to destabilize VirB11 more via targeting it to protease degradation (25) in order to inactivate the VirB T4SS earlier during the bacterium’s intracellular cycle.

The strongly reduced VirB T4SS activity observed by 24 h pi under our experimental conditions gave us the opportunity to evaluate the role of this secretion system in aBCV formation and bacterial egress, events which occur between 48 and 72 h pi (12). Bacteria displaying a loss of VirB T4SS activity by 24 h pi were significantly affected in their ability to remodel rBCVs into aBCVs, despite normal bacterial growth at these time points, and failed to undergo bacterial egress. These results clearly demonstrate a role of VirB type IV secretion in these late events of the *Brucella* intracellular cycle and argue that aBCV formation and bacterial egress are controlled by VirB T4SS effectors. aBCV formation requires a subset of host machineries involved in autophagy (12), suggesting that specific VirB T4SS effectors modulate this pathway to promote aBCV formation. Given the autophagic nature of these late vacuoles and their role in bacterial egress, it is also tempting to speculate that bacterial release via these organelles may invoke processes related to secretory autophagy which might be uncovered by identification of the VirB T4SS effectors controlling these processes.

## MATERIAL AND METHODS

### Bacterial strains and culture.

Wild-type *Brucella abortus* strain 2308 and its derivatives were grown on tryptic soy agar (TSA) (Difco) for 72 h at 37°C and subsequently in tryptic soy broth (TSB; Difco) at 37°C with shaking to an optical density at 600 nm (OD_600_) of ~1.0. When indicated, bacterial cultures were treated with 100 ng/ml of anhydrotetracycline (ATc; Acros Organics) 4 h prior to infection. All experiments performed with *B. abortus* strains, including the use of an ATc-inducible gene expression system which does not confer resistance to tetracycline, were carried out in a biosafety level 3 facility according to standard operating procedures approved by Washington State University Institutional Biosafety Committee and in compliance with NIH guidelines and CDC Division of Select Agents and Toxins regulations.

### Construction of *B. abortus* strain 2308Δ*virB11*::*virB11i*.

To generate a *B. abortus* strain in which the functionality of the VirB T4SS is controllable by the inducer ATc, we first deleted *virB11* within the *virB* chromosomal locus as follows: two DNA hemifragments flanking *virB11* were PCR amplified using either primers RC179 (5′-CGGTACCCGGGGATCCGAGAACCTGCAATGACACAG-3′) and RC180 (5′-GTCACTTCGGTTTGACATCAT-3′), or primers RC181 (5′-GTCAAACCGAAGTGACTGAAGCTGCAACTTTCACCC-3′) and RC182 (5′-ATGCCTGCAGGTCGACGCCAGTTGAAATAATCGTCGC-3′). The two hemifragments were fused via overlap extension PCR to generate a Δ*virB11* fragment containing an in-frame deletion of *VirB11* codons 6 to 356 while preserving the integrity of overlapping *virB10* and were cloned into the *sacB*-assisted pJC80 allelic replacement plasmid (15) using BamHI and SalI restriction sites to yield pJC80Δ*virB11*. pJC80Δ*virB11* was electroporated into wild-type *B. abortus* strain 2308, and merodiploids were selected for resistance to carbenicillin and sucrose sensitivity and subjected to sucrose selection, as previously described (15). *virB11* deletion at the correct chromosomal locus in *B. abortus* 2308Δ*virB11* was confirmed by PCR using primers RC189 (5′-GACGGAACCACAGTGCCAG-3′) and RC190 (5′-CGCCGATCATAACGACAAC-3′). To generate the complementing transposon mini-Tn*7*KΩ*tetRA-HA-virB11*, we first constructed the artificial bicistronic operon *P_aphA3_-dsred_m_-aphA3* by amplifying (i) the 1,050-bp *P_aphA3_-dsRed_m_* fragment from pJC44(13) using primers TW563 (5′-CAACTGCAGCCAGCGAACCATTTGAGG-3′) and TW575 (5′-TGCGAAACGATCCTCTATCGCGGCCGCTCTACTGG-3′) and (ii) the 800-bp *aphA3* gene from pBBR1MCS-2 using primers TW568 (5′-AGGATCGTTTCGCATGATTG-3′) and TW569 (5′-GCGGGATCCTCAGAAGAACTCGTCAAGAAG-3′). These fragments were then fused via overlap extension PCR using primers TW563 and TW569 and then cloned into pCR2.1TOPO (Thermo Fisher Scientific) according to the manufacturer’s instructions. The 1,850-bp *P_aphA3_-dsred_m_-aphA3* fragment was sequenced, verified, and then amplified using primers TW600 (5′-CTTCTCGAGGAATTCCCAGCGAACCATTTGAGG-3′) and TW598 (5′-CTCACTAGTGGATCCTCAGAAGAACTCGTCAAGAAG-3′) and cloned into pUC18T-miniTn*7* (26) using an In-Fusion cloning kit (Clontech) to generate pUC18T-miniTn7K-*dsRed*. Separately, a 1,107-bp *virB11* fragment was amplified from *B. abortus* 2308 genomic DNA (gDNA), subjected to HA tagging at its 5′ end using primers RC203 (5′-ATGTATCCATATGATGTTCCAGATTATGCTATGTCAAACCGAAGTGAC-3′) and RC164 (5′-GCCCAAGCTTCTCGAGTCATTGCGTCTTCTCACTGTG-3′), and fused via overlap extension PCR to a 705-bp *tetR-P*_*tetA*_ fragment amplified from Tn*10* using primers TW75 (5′-TAGAACTAGTGGATCCTTAAGACCCACTTTCACA-3′) and TW76 (5′-TTCACTTTTCTCTATCACTG-3′). The resulting 1,812-bp *tetR-P_tetA_-HA-virB11* fragment, initially cloned into pBBR1MCS-2 BamHI-SalI restriction sites, was further PCR amplified using primers RC163 (5′-GGTTCGCTGGGAATTCTTAAGACCCACTTTCACA-3′) and RC164 (5′-GCCCAAGCTTCTCGAGTCATTGCGTCTTCTCACTGTG-3′) and cloned as an EcoRI-XhoI fragment into pUC18T-miniTn7K-*dsRed* to generate pUC18T-miniTn*7*K-*dsRed*Ω*tetRA-HA-virB11* (Fig. 1A), which was verified by sequencing. miniTn*7*K-*dsRed*Ω*tetRA-HA-virB11* was then inserted at the *att* Tn*7* locus in either *B. abortus* 2308Δ*virB11* or *B. abortus* 2308 via electroporation of its delivery plasmid and the helper plasmid pUC18T-Tn7-*tnp*, as previously described (20), to generate strain 2308*ΔvirB11*::*virB11*^i^ or strain 2308::*virB11i*, respectively, and the results were confirmed by PCR amplification of the transposon insertion locus.

### Mammalian cell culture and infection.

Murine bone marrow-derived macrophages (BMMs) were generated from bone marrow of female C57BL/6J mice (Envigo) (6 to 12 weeks old) and cultured in Dulbecco’s modified Eagle’s medium (DMEM; Corning) (1 g/liter glucose) supplemented with 10% fetal bovine serum (FBS; Life Technologies, Inc.) and 10% L-929 mouse fibroblast-conditioned medium at 37°C in 10% CO_2_, as previously described (13). Two days prior to infection, BMMs were seeded at 5 × 10^4^ (24-well plates) cells/well or 1 × 10^6^ (6-well plate) cells/well and then infected with *B. abortus* strains at the desired multiplicity of infection (MOI) via a 10-min centrifugation at 400 × *g* and 4°C to synchronize infection. Following 20 min of incubation at 37°C in 10% CO_2_, BMMs were washed five times with 37°C DMEM to remove extracellular bacteria and replenished with complete medium, and medium supplemented with 100 μg/ml gentamicin (Gibco) was added between 1 and 2 h postinfection to kill the remaining extracellular *Brucella* cells. Afterward, the culture medium was replaced every 24 h during infection. When indicated, 100 ng/ml ATc (Acros Organics) was added to the culture medium for the specified times postinfection.

### Immunofluorescence microscopy.

BMMs were seeded at 5 × 10^4^ cells/well on 12-mm-diameter glass coverslips in 24-well plates and infected with DsRed_m_-producing *B. abortus* strains at an MOI of 10. At specified time points, coverslips were washed three times in 1× phosphate-buffered saline (PBS) and fixed using 3% paraformaldehyde–PBS (pH 7.4) at 37°C for 20 min. Immunostaining was carried out as described previously (13) using rat anti-mouse LAMP1 antibody (clone 1D4B; developed by J. T. August and obtained from the Developmental Studies Hybridoma Bank [DSHB] developed under the auspices of the National Institute of Child Health and Human Development [NICHD] and maintained by the University of Iowa, Department of Biological Sciences, Iowa City, IA) (1:500 dilution) followed by either Alexa Fluor 488-conjugated donkey anti-rat secondary antibody (Invitrogen) (1:500) or Cyanin 5-conjugated donkey anti-rat secondary antibody (Jackson ImmunoResearch Laboratories) (1:500). Samples were mounted using Mowiol 4-88 mounting medium and observed using either a Leica DM4000 B LED epifluorescence microscope (Leica Microsystems, Inc.) for quantitative analysis or a Leica TCS SP8 confocal microscope (Leica Microsystems, Inc.) for confocal image acquisition. Representative confocal micrographs were acquired as 1,024-by-1,024-pixel images using a 63×/1.4 numerical aperture (NA) HC PL APO objective and assembled using Adobe Photoshop CS6.

### aBCV formation and reinfection assays.

To measure aBCV formation and reinfection events at 72 h pi (12), BMMs were cultured and infected as specified above, except that gentamicin (20 μg/ml) was added between 24 h and 72 h pi for quantification of aBCV formation or was added between 24 and 48 h pi and then removed to measure bacterial reinfection between 48 and 72 h pi. aBCV formation was measured by scoring the percentage of infected cells containing large LAMP1-positive bacterial vacuoles, as described previously (12). To quantify reinfection events, a secondary, uninfected population of BMMs was loaded with 2 mM Cell Tracker Green CMFDA (5-chloromethylfluorescein diacetate) (Life Technologies, Inc.) for 30 min at 37°C, washed three times with PBS, and harvested using TrypLE cell dissociation enzyme (TrypLE Express [phenol free]; Life Technologies, Inc.) and gentle scraping. Cell Tracker Green-labeled BMMs (5 × 10^4^ cells/well) were then added to infected BMMs (1:1 ratio) at 44 h pi in the presence of gentamicin (20 μg/ml), which was removed at 48 h pi to allow bacterial reinfection to occur. Samples were collected at 72 h pi, immunostained, and analyzed for infection of Cell Tracker Green-labeled BMMs.

### *Brucella* intracellular growth.

To measure bacterial growth within BMMs, cells were cultured in 24-well plates and infected with *B. abortus* strains at an MOI of 10 in triplicate wells per time point as described above. When needed, gentamicin (20 μg/ml) was added to cell culture media between 24 and 72 h postinfection to mitigate bacterial reinfection events (12). At each time postinfection, BMMs were washed three times in 1× PBS and lysed using 0.1% Triton X-100 in distilled water. Serial dilutions of lysates were plated in duplicate on TSA and grown for 2 days at 37°C for CFU enumeration.

### Analysis of VirB11 production and stability.

To measure HA-VirB11 production in bacteria, *B. abortus* 2308Δ*virB11*::*virB11i* was cultured in TSB to an OD_600_ of 0.2 and then *HA-virB11* expression was induced by addition of 100 ng/ml ATc for 4 h and protein samples equivalent to 1 × 10^9^ bacteria were collected for Western blot analysis. For determination of the HA-VirB11 half-life in liquid culture, chloramphenicol (Cm; 20 µg/ml) was added after 4 h of ATc induction to block protein synthesis, protein samples were generated at various times post-Cm addition, and aliquots equivalent to 1 × 10^8^ CFU were analyzed via Western blotting. To measure HA-VirB11 production in infected BMMs (seeded in 6-well plates at a density of 1 × 10^6^ BMMs/well), *B. abortus* 2308Δ*virB11*::*virB11i* was either induced with ATc (100 ng/ml) for 4 h prior to infection or induced at the time of infection either for the duration of the experiment or for 6 h only for HA-VirB11 stability experiments. An MOI of 500 was used in all experiments, except in analyzing HA-VirB11 stability, where MOIs were adjusted to yield similar CFU counts at all time points analyzed (MOIs of 500 for between 4 and 12 h pi, of 50 for 24 h pi, and of 10 for 36 and 48 h pi). Protein lysates were generated from 2 wells and CFU enumerated from individual wells in triplicate for each time point analyzed. For determination of the HA-VirB11 half-life in infected BMMs, ATc was removed after 6 h of induction through 5 washes with DMEM, chloramphenicol (20 µg/ml) was added, and protein samples were generated at various times post-Cm addition. For Western blot analysis, samples were lysed in 2× SDS-PAGE sample buffer (0.12 M Tris [pH 6.8], 10% glycerol, 3.4% SDS, 0.2 M dithiothreitol [DTT], 0.004% bromophenol blue) and incubated at 100°C for 10 min. Samples corresponding to equivalent CFU counts were resolved by SDS-PAGE, transferred onto nitrocellulose (GE Healthcare Life Sciences) or polyvinylidene difluoride (PVDF; Thermo Fisher Scientific) membranes via Western blotting, and probed using either mouse anti-HA:11 (clone 16B12; BioLegend) (1:10,000) for HA-VirB11 detection or mouse monoclonal anti-Omp19 Sc10 (gift from Axel Cloeckaert, INRA, France) (1:10,000) for detection of *B. abortus* Omp19 as a bacterial sample loading control, followed by horseradish peroxidase (HRP)-conjugated anti-mouse IgG secondary antibodies (Cell Signaling Technology, Inc.) (1:10,000). All antibodies were diluted in TBS-Tween (0.14 M NaCl, 0.02 M Tris [pH 7.6], 0.1% Tween 20)–5% skim milk. Western blots were then developed using Super Signal West Femto maximum-sensitivity substrate (Thermo Fisher Scientific) and imaged using a BioRad Chemi-Doc gel imaging system (BioRad), and representative figures were assembled using Adobe Photoshop CS6.

### TEM1 β-lactamase protein translocation assay.

The translocation of *Brucella* VirB T4SS effector-TEM1 fusion constructs was evaluated by detecting TEM1 β-lactamase activity in infected J774A.1 macrophage-like cells, as previously described (20, 27). Derivatives of pJC121 expressing TEM1 fusions to BspD, BspI, or BAB2_0654 (20) were transformed into the *B. abortus* Δ*virB11*::*virB11i* strain by electroporation, and production of the TEM1 fusions of the correct size was verified by Western blotting using an anti-β-lactamase antibody (Qed Bioscience, Inc.) (1:10,000). Bacteria were grown in TSB, and ATc was added 4 h prior to infection. J774A.1 cells seeded (5 × 10^4^/well) in 96-well glass bottom plates (Cellvis) were infected at an MOI of 1,000 in medium supplemented with 0.1 mM IPTG (isopropyl-β-d-thiogalactopyranoside) to induce expression of the TEM1 fusion constructs in the presence or absence of ATc (100 ng/ml). At 16 and 24 h pi, cells were incubated with fluorescent substrate CCF2-AM (LiveBLAzer FRET-B/G loading kit; Invitrogen) in β-lactamase loading solution supplemented with 15 mM probenecid (Invitrogen) in the dark for 90 min at room temperature, washed in 1× PBS containing 1 mM probenecid, and then observed using epifluorescence in imaging medium containing FluoroBrite DMEM (Gibco), 25 mM HEPES, 10% heat-inactivated FBS, and 2.5 mM probenecid, using a Leica DMI6000 Spinning Disk confocal microscope fitted with a β-lactamase Blue/Aqua 41031 filterset (Chroma Technology Corp.). At least 300 cells were counted in triplicate wells to determine the percentage of cells displaying a CCF2-AM fluorescence shift (TEM1 translocation positive).

### Quantitation of *virB* gene expression.

To measure expression of *virB* genes during infection, BMMs were seeded in 6-well plates and infected with wild-type *B. abortus* strain 2308 at a MOI of 200 (1, 4, 8, and 16 h pi), 100 (24 h pi), or 50 (48 h pi). At each time point, BMMs from 4 wells were collected into 1 ml of TRIzol reagent (Ambion; Life Technologies, Inc.) and homogenized using a FastPrep-24 instrument (MP Biomedicals) through 4 cycles of 45 s at 4,000 rpm. Total RNA and genomic DNA (gDNA) were isolated using recommended TRIzol extraction procedures. RNA samples were treated using a Turbo DNA-free kit (Ambion) to remove DNA contaminants and subjected to reverse transcription using a Maxima First Strand cDNA synthesis kit (Thermo Fisher Scientific). Quantitative real-time PCR (RT-PCR) was performed using Luminaris Color HiGreen qPCR master mix (Thermo Fisher Scientific) and a Bio-Rad CFX96 Touch real-time PCR detection system. The indicated primers were used for quantification of either *virB4* (*virB4-*f [5′-GGCGCTCAATCCAAATACGCG-3′] and *virB4-r* [5′-GTAATCGCTATACATCAGGCC-3′]) or *virB11* (*virB11-f* [5′-TGACTTTCGAGCGGCTGG-3′] and *virB11-r* [5′-GCACGACGACATCCACCA-3′]) mRNAs. Mean *n-*fold expression levels of cDNA from three independent biological replicates, each measured in duplicate, were normalized to gDNA levels and calibrated according to the threshold cycle (2^−Δ*CT*^) method (28).

### Statistical analysis.

Statistical analysis was performed using GraphPad Prism 6 software. All results are presented as means ± standard deviations (SD) of results from at least three independent experiments, unless otherwise stated. Statistical significance was determined either using an unpaired, two-tailed Student’s *t* test or, for group analysis, using two-way analysis of variance (ANOVA) followed by either Bonferroni’s or Sidak’s multiple-comparison tests. A *P* value of <0.05 indicates a statistically significant difference.

## SUPPLEMENTAL MATERIAL

Figure S1  Gentamicin prevents *Brucella* reinfection events in BMMs. Representative confocal micrographs show reinfection events in BMMs infected with *B. abortus* 2308Δ*virB11*::*virB11i* either upon sustained ATc treatment (ATc^72h^) or following 4 h of ATc preinduction (ATc^−4h^) and sustained gentamicin (Gm) treatment between 48 and 72 h pi. Cell Tracker Green-labeled BMMs appear in green, bacteria in red, and LAMP1-positive compartments and aBCVs (ATc^72h^) in blue. Note that cell Tracker Green-labeled BMMs do not contain bacteria. Scale bar, 20 µm. Download Figure S1, TIF file, 1.3 MB
