# Supplementary figures and images for: Postreplication Roles of the Brucella VirB Type IV Secretion System Uncovered via Conditional Expression of the VirB11 ATPase
Source: mBio. 2016 Nov 29;7(6):e01730-16. doi: 10.1128/mBio.01730-16 (PMC5137499; doi:10.1128/mBio.01730-16)

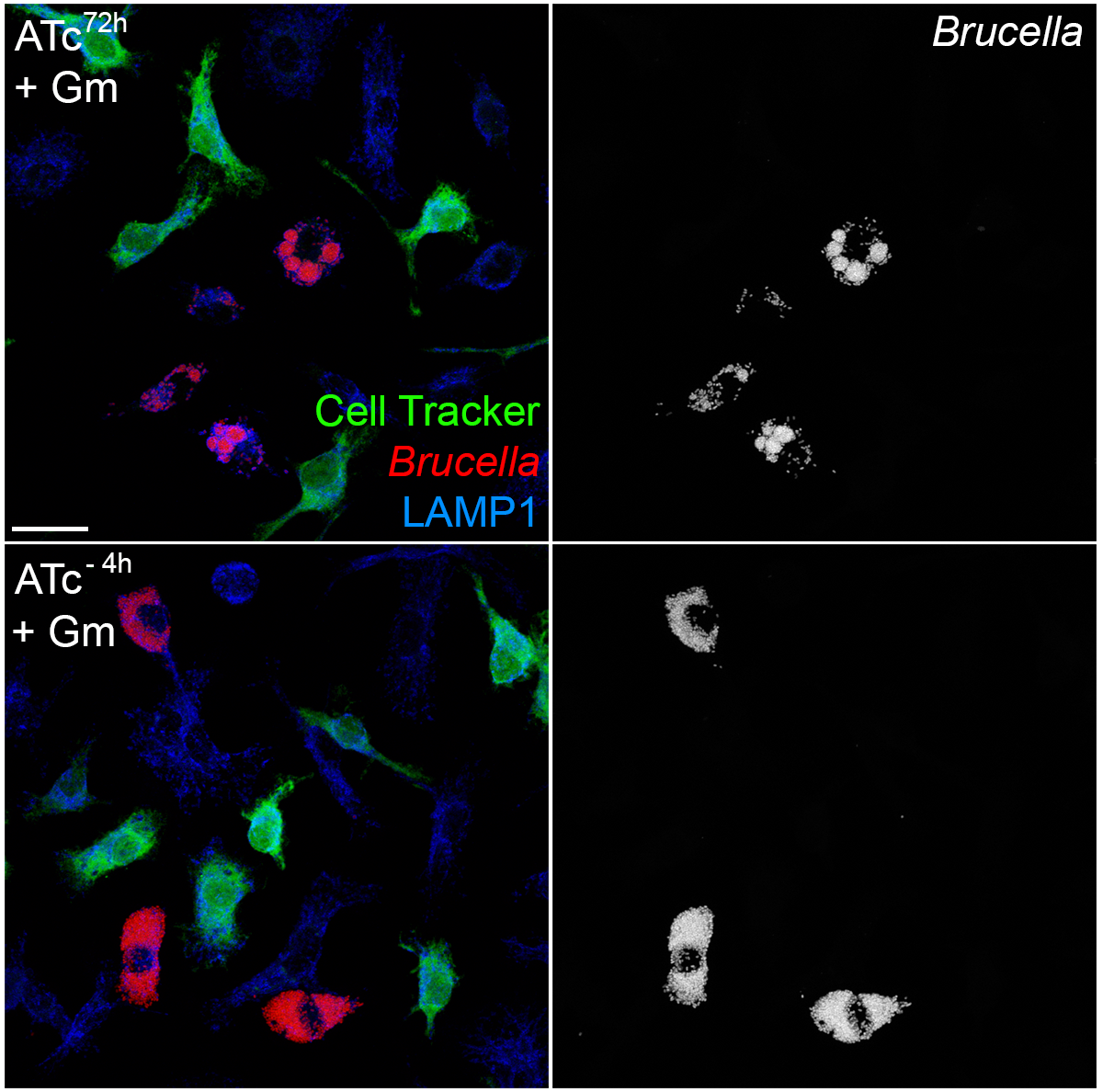

Supplement: Figure S1 — Gentamicin prevents Brucella reinfection events in BMMs. Representative confocal micrographs show reinfection events in BMMs infected with B. abortus 2308ΔvirB11::virB11i either upon sustained ATc treatment (ATc72h) or following 4 h of ATc preinduction (ATc−4h) and sustained gentamicin (Gm) treatment between 48 and 72 h pi. Cell Tracker Green-labeled BMMs appear in green, bacteria in red, and LAMP1-positive compartments and aBCVs (ATc72h) in blue. Note that cell Tracker Green-labeled BMMs do not contain bacteria. Scale bar, 20 µm. Download [file mbo006163090sf1.tif]
